# Prognostic Role of Red Cell Distribution Width and Other Routine Clinico-Pathological Parameters in Dogs with Acute Pancreatitis

**DOI:** 10.3390/ani12243483

**Published:** 2022-12-09

**Authors:** Carlo Guglielmini, Paolo Emidio Crisi, Antonio Maria Tardo, Roberta Di Maggio, Barbara Contiero, Andrea Boari, Federico Fracassi, Arianna Miglio

**Affiliations:** 1 Department of Animal Medicine, Production and Health, University of Padua, Viale dell’Università 16, 35020 Padova, Italy; 2 Department of Veterinary Medicine, University of Teramo, Piano d’Accio, 64100 Teramo, Italy; 3 Department of Veterinary Medical Sciences, University of Bologna, Via Tolara di Sopra 50, 40064 Ozzano Emilia, Italy

**Keywords:** canine, laboratory biomarker, acute pancreatitis, outcome, RDW, RDW-to-total calcium ratio, neutrophils-to-lymphocytes ratio, platelets-to-lymphocytes ratio

## Abstract

**Simple Summary:**

This study aimed to assess the prognostic value of some commonly used and inexpensive hematological and biochemical parameters in dogs with acute pancreatitis. This is a multicenter study including 70 client-owned dogs. Statistical analysis was performed to obtain the accuracy of clinical and laboratory variables in order to predict short-term death (i.e., dead by 14 days) and to identify independent predictors of death. The survival rate was 72.9% (51 dogs) and 19 dogs died within 14 days of admission from AP. Red Cell Distribution Width (RDW) and blood urea nitrogen (BUN) had good accuracy to predict short-term death with the cut-off of >12.7% and >42 mg/dL, respectively. The results indicate that RDW, BUN and bilirubin are useful predictors of short-term death in dogs with acute pancreatitis.

**Abstract:**

This study aimed to assess the prognostic value of red cell distribution width (RDW) RDW-to-calcium ratio (RDW/Ca), neutrophils-to-lymphocytes ratio (N/L), platelets-to-lymphocytes ratio (P/L) and other easy to obtain and inexpensive hematological and biochemical parameters in dogs with acute pancreatitis. This is a multicenter, retrospective cohort study including 70 client-owned dogs. The accuracy of clinical and laboratory variables to predict short-term death (i.e., dead by 14 days) was tested by calculating the area under the receiver-operating characteristic curve (AUC). Independent predictors of death were identified using the multivariable Cox proportional hazards regression model. The survival rate was 72.9% (51 dogs) and 19 dogs died within 14 days of admission from AP. RDW and blood urea nitrogen (BUN) had good accuracy to predict short-term dead with AUC of 0.74 and 0.70 at the cut-off of >12.7% and >42 mg/dL, respectively. According to the multivariable model, RDW (hazard ratio and 95% confidence interval [HR, 95% CI] = 5.08, 95% CI = 1.14–22.67; *p* = 0.03), BUN (HR = 1.00, 95% CI = 1.00–1.01; *p* < 0.01) and bilirubin (HR = 2.46, 95% CI = 1.38–4.39; *p* < 0.01) were independent predictors of death. The results indicate that RDW, BUN and bilirubin are useful predictors of short-term death in dogs with acute pancreatitis.

## 1. Introduction

Canine acute pancreatitis (CAP) is a severe and life-threatening inflammatory disease with a reported death rate of up to 58%. This condition is commonly diagnosed in dogs and hospitalization length can vary between hours and weeks [1,2,3]. The early assessment of disease severity and the prediction of clinical outcomes for CAP has been postulated as fundamental in order to improve treatment approach and to reduce mortality [4,5,6]. Nevertheless, to date, the real estimation of prognosis in CAP remains challenging [1,2,3,5].

As in human beings with acute pancreatitis (AP), several potential laboratory prognostic markers have been proposed for CAP. In particular, different serum markers, such as C-reactive protein (CRP) [3,7], CRP-to-albumin ratio [8], canine pancreatic lipase (cPL), and specific canine pancreatic lipase-(Spec cPL) [2,9,10], 1,2-o-dilauryl-rac-glycero-3-glutaric acid-6-methylresorufin ester [DGGR] lipase [10,11], azotemia (i.e., serum urea and creatinine) [1,12,13], and asymmetric dimethylarginine [14], as well as some urinary markers, namely urine protein-to-creatinine ratio [15], urinary GGT-to-creatinine ratio [15], and plasma coagulative factor antithrombin [9], have been studied with debatable prognostic results. Furthermore, many of these parameters can be variably expensive, are time consuming and not rapidly available at patient admission.

On the other hand, some simple, independent, economic and non-invasive hematological parameters, promptly and commonly available at patient presentation, seem to be crucial for the early identification of disease severity and mortality in people with AP. Among them, red cell distribution width (RDW), RDW-to-total calcium ratio (RDW/Ca), neutrophils-to-lymphocytes ratio (N/L) and platelets-to-lymphocytes ratio (P/L) have been powerfully associated with negative outcome in people with AP [16,17,18,19,20,21]. At present, some observational studies have investigated the role of RDW in dogs with different systemic disorders, including cardiovascular disorders (myxomatous mitral valve disease and pulmonary hypertension) [22,23,24,25], acute trauma, heartworm disease, and dogs admitted to intensive care unit or hospitalized for medical and surgical diseases [26,27,28,29,30], providing conflicting results. The prognostic value of RDW and RDW/Ca has been poorly investigated in dogs with AP. Moreover, the diagnostic and prognostic significance of N/L and P/L have been investigated in some studies of dogs with septic peritonitis and systemic inflammatory response syndrome [31,32], and, only in one study, in dogs and cats with AP [6]. In these studies, the ability of these biomarkers to estimate disease course and outcome, even if promising, seems controversial and requires additional studies.

This study investigates the prognostic role of RDW, RDW/Ca, N/L and P/L, in addition to other routine clinico-pathological parameters, as biomarkers in CAP. We hypothesized that the increase in these parameters can be associated with the negative outcome in dogs with AP.

## 2. Material and Methods

### 2.1. Study Design and Inclusion Criteria

This is a retrospective cohort study conducted at three veterinary teaching hospitals (VTHs), from January 2019 to December 2021. The study was approved by the management committee of the VTH of the University of Padua and all owners gave informed, written consent for all investigations. The inclusion criteria were: (1) definitive diagnosis of AP based on consistent history and clinical signs, namely acute onset of one or more compatible clinical signs including anorexia, abdominal pain, vomiting and lethargy; (2) specific laboratory analyses, including screening tests for CAP (i.e., abnormal result of semi quantitative SNAP cPL test, corresponding to a cPL > 200 ug/L, or DGGR lipase > 141 IU/L) associated with confirmatory tests (Spec cPL concentrations between 201–399 ug/L considered equivocal) or solely confirmatory tests (Spec cPL > 400 ug/L considered consistent with a diagnosis of pancreatitis); (3) results of ultrasonographic examination performed within 48 h from admission consistent with AP (i.e., thickened hypoechoic pancreas with blurred margins, surrounded by hyperechoic adipose tissue and presence of free peritoneal fluid); (4) availability of CBC and serum biochemical profile within 24 h from admission; and (5) availability of follow-up information for at least 35 days [1,2,33].

Dogs were excluded from the study if clinical signs were present for more than seven days or if previous episodes consistent with AP were reported.

### 2.2. Data Collection

The data extracted from the electronic internal medical database included signalment, history, physical examination findings at admission, results of CBC, biochemistry profile and abdominal ultrasonographic examination obtained within 24 h from admission, any previously administered drug (dichotomized as treatment vs. no treatment) and presence of concurrent diseases (dichotomized as present vs. absent) and follow up information. Regarding outcomes, the dogs were dichotomized into two groups: non-survivors (dogs that died in the period of 14 days from admission) and survivors (dogs still alive 14 days after admission). The survival data were derived from databases of each VTH or through telephone interview with the dogs’ owners.

### 2.3. Laboratory Findings

CBC and biochemistry profiles were performed using routine methods at diagnostic laboratories of the three VTHs. The hematological variables, red blood cell (RBC), RDW, hematocrit (Hct), hemoglobin (Hgb), mean corpuscular volume (MCV), white blood cell (WBC), neutrophils, lymphocytes, platelets and mean platelets volume (MPV), were measured in all three centers using an automated CBC analyzer (Advia 120, Hematology system, Siemens, Munich, Germany) previously validated for canine hematology [34,35,36]. The biochemical parameters, blood urea nitrogen (BUN), creatinine, total proteins, albumin, total bilirubin, serum alkaline phosphatase (SAP), alanine aminotransferase, Ca, phosphate, glucose, triglycerides, cholesterol and CRP, were measured using AU analyzers (AU400, Beckman Coulter/Olympus, Brea, CA, USA; AU480, Beckman Coulter/Olympus, Brea, CA, USA). Internal quality controls provided by manufacturers were run daily for hematology and clinical biochemistry analysis. The external quality controls were performed for all analyzers using human control material every week.

The rapid semi quantitative SNAP cPL test (Idexx Laboratories) was performed in house according to the manufacturer’s instructions and the Spec cPL was measured in a commercial laboratory (Idexx Laboratories, Inc.), both using an ELISA method. The DGGR lipase activity was measured using a commercially available assay (Diazime Laboratories, AU480; Beckman Coulter chemistry analyzer) according to the manufacturer’s instructions.

### 2.4. Statistical Analyses

One open source (R Core Team 2022. R: A language and environment for statistical computing. R Foundation for Statistical Computing, Vienna, Austria. https://R-project.org/ accessed on 17 March 2020) and different commercially available software’s (SAS version 9.3, SAS Institute Inc., Cary, NC, USA; MedCalc version 12.6.1.0, MedCalc Software, Ostend, Belgium) were used for statistical analyses. Demographic and clinical characteristics included: sex; breed; age; body weight (BW); presence of concurrent diseases and ongoing treatment at the time of inclusion. For sex, breed, concurrent diseases and ongoing treatment, the following binary categories were considered, respectively: male and female; purebred and crossbred; and yes and no. The following continuous CBC and biochemical variables were considered: RBC, WBC, platelets, neutrophils, lymphocytes, N/L, P/L, Hct, Hgb, MCV, MPV, RDW, and serum total protein, albumin, BUN, creatinine, bilirubin, cholesterol, triglycerides, glucose, SAP, alanine-amino transferase, Ca, phosphate and CRP and the RDW/Ca.

The continuous data were assessed using the Shapiro-Wilk’s test for normality and reported as mean and standard deviation (SD) or median and minimum and maximum for normally and not-normally distributed data, respectively. Categorical variables were reported as number and percentage. Dogs dead within 14 days from admission (i.e., non-survivors) were considered to have died as a result of AP if no other severe concomitant diseases were detected. Comparison of clinical and laboratory variables between non-survivors and survivors was conducted using the Student’s *t*-test or the Mann-Whitney test for normally or not-normally distributed continuous variables, respectively, and the z-test for categorical variables. The association between all laboratory variables and the investigated variables RDW, RDW/Ca, N/L and P/L was assessed using Spearman rank correlation analysis.

The predictive ability of variables showing significant differences in the aforementioned analyses to distinguish between non-survivors and survivors was evaluated by receiver operating characteristic (ROC) curve analysis. The corresponding area under the curve (AUC) with 95% confidence interval (CI), and sensitivity and specificity were calculated at different cut-off points. Youden criterion was used to calculate thresholds that maximize the sensitivity and specificity of the method.

Univariate and multivariable regression with Cox models was performed to determine whether a significant relationship existed between clinical (i.e., age, body weight, sex, breed, presence of concurrent diseases and previous treatment) and laboratory variables and survival. The endpoint was death within 14 days from admission. The hazard ratio (HR) and 95% CI were calculated considering one-unit change or reference level for continuous or categorical variables, respectively. Investigated variables RDW and RDW/Ca significantly predictive of the outcome (i.e., death within 14 days) in the ROC curve analysis were included in the univariate model, either as a continuous or binomial factor, dichotomized based on above calculated threshold. As numerous variables were considered in the univariate analysis (i.e., six clinical and 26 laboratory variables, respectively), correction for multiple testing was applied. In particular, alpha risk inflation as a result of multiple testing was addressed by calculating the Q value, which is preferable to the *p* value to control both the positive false discovery rate (pFDR) and FDR [37]. Thus, only variables with Q < 0.05 in the univariate analysis were included in the final multivariable Cox proportional hazards regression model with the stepwise selection method.

A value of *p* < 0.05 was considered significant for all analyses.

## 3. Results

### 3.1. Study Population and Laboratory Parameters

This study included 70 dogs with AP (45, 13 and 12 dogs from the University of Bologna, Padua and Teramo, respectively): 48 females (68.6%), of which 32 were spayed; and 22 males (31.4%), of which eight were neutered. The mean age was 10 ± 4 years (mean ± SD) and the BW was 14 ± 11 kg (mean ± SD). Common breed included mixed breed (33 dogs, 47.1%), Miniature Pinscher (four dogs, 5.7%), Yorkshire Terrier, Dachshund and Jack Russell Terrier (three dogs, 4.3%) and Poodle, Beagle, Boxer, Golden Retriever, Maltese and Rottweiler (two dogs, 2.9%).

At admission, 48 dogs (69%) had one or more concurrent extra-pancreatic diseases, including 12 dogs (17%) with endocrine diseases (diabetes mellitus in seven cases, hypothyroidism in two cases, hyperadrenocorticism in two cases and hypoadrenocorticism in one case), 10 dogs (14%) with neoplastic diseases (lymphoma three cases, hepatic neoplasia and insulinoma one case each and undetermined neoplasia five cases), nine dogs (13%) with renal and lower urinary tract diseases (chronic kidney disease six cases, lower urinary tract infection two cases and nephrotic syndrome one case), eight dogs (11%) with gastrointestinal diseases (chronic enteropathy seven cases and gastritis one case), five dogs (7%) each with epilepsy or myxomatous mitral valve disease, two dogs each with prostatic disease, immune-mediated polyarthritis or leishmaniosis and one dog each with internal otitis, blunt trauma, immune-mediated thrombocytopenia, septic peritonitis or orthopedic disease. Among these, 35 dogs (50%) were receiving medical treatment for the underlying disease. Regarding treatment after the diagnosis of AP, all dogs, with the exception of one, who died on the day of diagnosis, received a combination of two or more treatments. In particular, the most used therapies included intravenous fluid therapy in 65 (93%) dogs, antiemetics in 62 (89%) dogs, gastrointestinal protectants (i.e., proton pump inhibitors or histamine type-2 receptor antagonists) in 55 (79%) dogs and analgesics in 48 (69%) cases. Additional treatments comprised a commercial or homemade low-fat diet, probiotics, antithrombotics, antibiotics, prokinetics, N-acetyl cysteine, frozen plasma, insulin, glucocorticoids and ursodeoxycholic acid.

After a minimum available follow-up time of 37 days, 19 dogs (27.1%) were non-survivors (median 4 days, range 1–13), while 51 dogs (72.9%) were survivors (median 140 days, range 37–1042). Table 1 presents a comparison of clinical and laboratory variables between non-survivors and survivors. The median BUN, RDW, and RDW/Ca were significantly higher in non-survivors compared to survivors dogs (*p* = 0.02, *p* = 0.01 and *p* = 0.03, respectively), whereas mean RBC was significantly lower (*p* = 0.02).

Table 2 shows the results of the Spearman’s correlation of the laboratory variables with RDW, RDW/Ca, N/L and P/L. All of the significant correlations that were found, excluding those between linked variables (e.g., RDW and RDW/Ca), were weak (r < 0.50). Serum phosphate, SAP, WBC, neutrophils count, MPV, and N/L were positively correlated with RDW. White blood cells, neutrophils count and MPV were positively correlated with RDW/Ca, whereas glucose and hematocrit were negatively correlated with RDW. Serum cholesterol, triglycerides, SAP, and P/L were positively correlated with N/L. Furthermore, serum protein, albumin, cholesterol, glucose and SAP were positively correlated with P/L.

**Figure 1:**
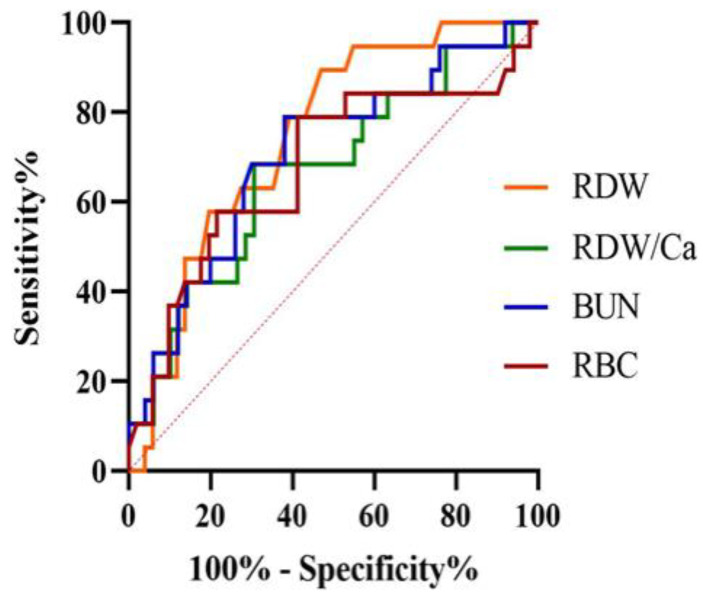
Receiver operating characteristic curve for red cell distribution width (RDW), RDW-to-total serum calcium ratio (RDW/Ca), blood urea nitrogen (BUN) and red blood cells count (RBC) for the prediction of death within 14 days in a population of 70 dogs with acute pancreatitis. The area under the curve was 0.74 (95% confidence interval = 0.63–0.84) for RDW at the cut-off point >12.7%.

Table 3 and Figure 1 present the results of the ROC curve analyses. Red cell distribution width > 12.7% (AUC = 0.74, 95% CI 0.63–0.84) and BUN > 42 mg/dL (AUC = 0.70, 95% CI 0.58–0.81) were the optimal cut-offs to discriminate non-survivors from survivor dogs.

### 3.2. Univariate and Multivariable Regression Analysis

Univariate logistic regression showed that death within 14 days was correlated with serum BUN (*p* < 0.01), creatinine (*p* = 0.01), bilirubin (*p* < 0.01), phosphate (*p* < 0.01), SAP (*p* = 0.03), RBC (*p* = 0.03), RDW (*p* = 0.01), RDW > 12.7% (*p* < 0.01), RDW/Ca (*p* = 0.02) and RDW/Ca > 1.4 (*p* < 0.01) (Table 4).

The final multivariable model was built, including all predictors, with Q < 0.05 in the univariate model and class RDW > 12.7% was used instead of RDW/Ca > 1.4 to avoid multi-collinearity. The model confirmed that increased BUN (HR = 1.00, 95% CI = 1.00–1.01; *p* < 0.01), bilirubin (HR = 2.46, 95% CI = 1.38–4.39; *p* < 0.01) and RDW > 12.7% (HR = 5.08, 95% CI = 1.14–22.67; *p* = 0.03) were significant predictors of negative outcome (Table 5).

## 4. Discussion

This study evaluated the prognostic usefulness of some simple and inexpensive laboratory parameters to predict short-term death in dogs with AP. The main results were the identification of the cut-off value of RDW with good accuracy to predict death within 14 days in these dogs. Furthermore, in addition to BUN and bilirubin, RDW can be an independent predictor of the negative outcome in the same animals.

Acute pancreatitis in dogs is challenging for veterinary clinicians due to its complex inflammatory response, multifaceted clinical presentation associated with local and multisystem complications and high mortality rate [2,3,9,33]. Therefore, the identification of factors associated with negative outcome is an important advancement in the awareness of this life-threatening disease. Preliminary analysis of the results of the present study showed that RDW, RDW/Ca and BUN, but not N/L and P/L, were significantly increased in non-survivors compared to survivors. Our results are in agreement with other studies that identified significantly increased concentrations of serum renal function biomarkers (i.e., BUN and creatinine) between non-survivors and survivors dogs with AP [7,12,13]. Azotemia has been associated with the increased risk of mortality and acute kidney injury has been predicted to be a comorbidity in dogs with CAP [7,12]. Only phosphate, but not BUN, was positively but weakly correlated with RDW in dogs in the present study. Furthermore, no correlation was found between RDW and RDW/Ca, and RBC, Hb and Hct, with the only exception of weak negative correlation between RDW and Hct. The association between RDW and regenerative anemia has been demonstrated in previous studies on dogs with various systemic and cardiovascular disorders [22,23,24,25,26,27,28,29]. Our findings suggest that the increased RDW and RDW/Ca in non-survivor dogs were not correlated to anemia and confirmed that anemia was not associated with a negative prognosis in dogs with AP [1,2,3,7].

The results of the ROC curve analyses demonstrated the useful prognostic role of RDW, BUN and, to a lesser extent, of RDW/Ca, but not of N/L and P/L. In particular, cut-off values of >12.7% and >1.4 for RDW and RDW/Ca had an accuracy of 0.74 and 0.67, respectively, to predict death within 14 days in dogs with AP. Regarding RDW, sensitivity was quite excellent (89%), but specificity was relatively low (53%).

The results of the univariate logistic regression analysis failed to identify an association between clinical parameters and the negative outcome. In particular, previous drug administration and the presence of concurrent disease did not impact the outcome. On the other hand, different laboratory parameters were significantly associated with short-term death, including BUN, creatinine, bilirubin, phosphate, SAP, RBC, RDW and RDW/Ca. However, only bilirubin and RDW > 12.7% were retained in the final multivariable model that was carried out using a direct approach to avoid both pFDR and FDR. In particular, dogs with RDW > 12.7% had a fivefold risk of mortality compared to those with RDW ≤ 12.7%. Similar results were obtained when RDW/Ca > 1.4 was used in the multivariable model instead of RDW > 12.7% (data not shown). Two recent studies reported the relationship between increased RDW and all-cause mortality in dogs with myxomatous mitral valve disease and those hospitalized for different disorders [25,29]. Conversely, RDW did not predict all-cause mortality in dogs with trauma and those admitted to the intensive care unit of a referral veterinary hospital for miscellaneous disorders [26,30]. In people, numerous retrospective studies, employing different methodologies, demonstrated that admission RDW significantly predicts clinical outcome and mortality in patients with AP, and suggests that this parameter is a strong prognostic factor of both severity and mortality for AP [19,38,39,40]. Interestingly, the cut-off values identified in dogs of the present study were slightly lower than those previously found predictable of mortality (i.e., >14.0% and >1.7 for RDW and RDW/Ca, respectively), but similar if not the same as those predictable of severity (i.e., >13.0% and >1.4 for RDW and RDW/Ca, respectively) in people with AP ^21^. Although the underling pathophysiologic mechanism relating RDW and prognosis in people with AP is unclear, it has been postulated that it could be partially mediated by stress and inflammation responses [41]. In people with AP, an increase in some inflammatory cytokines, such as tumor necrosis factor alpha, interleukin-1 and interleukin-6 due to sepsis, have been shown to contribute to RDW elevation by reducing RBC survival and maturation in the bone marrow, increasing the release of newer and larger reticulocytes in the peripheral blood [19,20,42]. These results overlap with those found in the canine species [9]. Thus, RDW is suggested to reflect the degree of systemic inflammatory response that occurs in AP, and is useful to predict its severity and outcome [21]. Regarding RDW/Ca ratio, it has been proven to be an excellent predictor of AP severity and mortality in people [43]. The association between hypocalcemia within the first 24 h and AP severity seems to be caused by calcium soap formation [43], and hypocalcemia can be a concern in dogs with AP [44].

The reference ranges of hematological variables, including RDW, can vary for different laboratories and the harmonization of red cell sizing is an issue in both human and veterinary hematology [35,45]. Indeed, despite the RDW cut-off of 12.7% identified in the present study falls within the reference intervals of the used instruments, the results suggest that some individuals with increased RDW values, even if within the reference intervals, may have a negative outcome, as already observed in dogs with myxomatous mitral valve disease [25].

Regarding N/L and P/L, this study did not demonstrate their prognostic usefulness in dogs with AP. These results are complementary to those of previous studies showing no significant relationship between N/L or P/L and disease severity, despite finding an increase in dogs with AP compared with healthy animals [6]. Our study contributes and underlines that, in veterinary medicine, there is a greater need for more research regarding these ratios, as conclusions obtained on their use as prognostic markers in cases of severe systemic inflammatory processes (e.g., AP, septic peritonitis, and systemic inflammatory response syndrome) are different [6,31,32,46]. Conversely, in human medicine, N/L and P/L ratios are extensively used as useful inflammatory markers in several diseases. Elevated N/L and P/L at admission have been confirmed as simple hematological predictors of adverse outcome in patients with AP [20,47]. The correlation between these ratios and disease prognosis seems to be related to the role of these inflammatory cells in the production and secretion of pro-inflammatory cytokines. Neutrophils activate the non-specific immune reaction and play a fundamental role in the pathogenesis of AP; on the other hand, T-helper lymphocytes decline significantly within 6 h of the onset of AP [20]. Moreover, platelets also seem to be able to phagocytize, interact and modulate the leukocyte function by secreting cytokines [48]. Interestingly, increased P/L, more so than N/L, seems to be associated with a prolonged course of CAP [6].

The use of different biochemistry analyzers, even from the same manufacturer, could represent a limitation of the study. However, AU analyzers have a proven ability to provide consistent results for precision, linearity/analytical measurement range, method comparison and reference ranges from different and separate laboratories [49]. Therefore, data obtained using these analyzers from multiple institutions could be merged for aggregate analysis [49].

Due to its retrospective design, this study has different inherent limitations. First, some potential confounding factors should have been considered, including unrecognized deficiency of iron, vitamin B12 and folates, hemolysis and anemia [19]. Nevertheless, the results of the present and others studies have demonstrated that anemia is not correlated with increased RDW and RDW/Ca and with negative outcome in dogs with AP [1,2,3,7]. Second, the treatment of the enrolled dogs was not standardized and followed specific requirements for each animal at attending clinician discretion. However, treatments performed in most of the dogs (i.e., intravenous fluids, analgesia and anti-emesis) followed suggested indications for the management of CAP [50]. A further limitation could be related to the presence of concurrent diseases that potentially affect the outcome. Indeed, it is reasonable that the presence of underlying extra-pancreatic disorders may negatively influence prognosis. However, it is worth noting that the statistical analysis showed no association between comorbid conditions and short-term death in the dogs of the present study. The small number of cases has been included and the relatively low number of deaths could have limited our capability to precisely assess the accuracy of the studied parameters in predicting mortality in our patient cohort. However, the mortality rate in dogs of the present study (27.1%) is similar to that previously found by other authors [1,9]. Finally, the diagnosis of AP was not confirmed by histopathological examination, but commonly accepted clinical diagnostic tests were used.

## 5. Conclusions

The present study supports the hypothesis that RDW and associated RDW/Ca, in addition to BUN and bilirubin, are useful, non-invasive, and accurate predictors of short-term mortality in dogs with AP. Thus, these inexpensive laboratory parameters can be used for the early identification of animals with an elevated risk of fatal AP needing prompt treatment in specialized intensive care units and for refining the prognosis in affected animals. Conversely, this study was not able to demonstrate that N/L and P/L have prognostic utility in dogs with AP. Further prospective studies with a larger sample size of dogs and examining various outcomes related to CAP are needed to confirm these promising results.

## Figures and Tables

**Table 1:** Clinical and laboratory data for the total study cohort and comparisons between non-survivors and survivors in 70 dogs with acute pancreatitis. Continuous variables were expressed as mean standard deviation (age and bodyweight) or as median with minimum and maximum in brackets (laboratory data).

Variable	Total Cohort (*n* = 70)	Non-Survivors (*n* = 19)	Survivors (*n* = 51)	*p* Value
Age (years)	10 ± 4	10 ± 4	10 ± 4	0.98
Weight (kg)	14 ± 11	11 ± 5	15 ±12	0.11
Sex (F/M)	48/22	14/5	34/16	0.79
Purebred (%)	27 (53%)	7 (37%)	30 (59%)	0.17
Concurrent diseases (%)	48 (69%)	15 (79%)	33 (65%)	0.39
Previous treatment (%)	35 (50%)	12 (63%)	23 (45%)	0.28
CRP (mg/dL)	4.9 (0.0–55.4)	3.8 (0.0–36.7)	5.7 (0.3–55.4)	0.44
BUN (mg/dL)	42 (15–734)	66 (17–734)	39 (15–267)	**0.02**
Creatinine (mg/dL)	1.0 (0.4–14.3)	1.0 (0.4–14.3)	1.0 (0.5–7.5)	0.27
Total protein (g/L)	64.9 (34.9–98.0)	68.0 (34.9–90.0)	64.6 (38.2–98.0)	0.48
Albumin (g/L)	29.2 (8.2–39.0)	29.9 (8.2–39.0)	27.3 (18.7–37.6)	0.14
Bilirubin (mg/dL)	0.20 (0.01–3.20)	0.30 (0.01–3.20)	0.2 (0.1–1.2)	0.05
Cholesterol (mg/dL)	280 (108–779)	277 (147–779)	285 (109–663)	0.59
Triglycerides (mg/dL)	95 (28–1090)	95 (33–1090)	98 (28–704)	0.43
Calcium (mg/dL)	9.7 (7.0–13.7)	9.6 (7.0–13.7)	9.7 (8.0–11.6)	0.90
Phosphate (mg/dL)	4.7 (2.2–29.0)	5.3 (2.9–29.0)	4.5 (2.2–15.2)	0.17
Glucose (mg/dL)	97 (11–893)	100 (11–618)	96 (37–893)	0.89
ALT (U/L)	93.5 (11–11463)	107 (19–1988)	86 (11–11463)	0.23
SAP (U/L)	401 (18–10664)	410 (18–10246)	399 (26–10664)	0.23
RBC (10^6^/μL)	6.3 (2.6–10.3)	6.2 (3.6–10.3)	6.6 (3.9–8.8)	**0.02**
Hemoglobin (g/dL)	15.0 (8.1–24.7)	14.9 (8.1–24.7)	15.0 (8.6–22.0)	0.19
Hematocrit (%)	42.7 (23.8–61.9)	42.1 (24.0–61.9)	43.3 (23.8–61.8)	0.08
MCV (fL)	68.5 (54.3–81.0)	69.8 (54.3–81.0)	67.2 (56.1–80.0)	0.07
WBC (10^3^/μL)	15.3 (4.5–56.3)	16.7 (5.0–56.3)	13.1 (4.5–41.3)	0.53
Neutrophils (10^3^/μL)	12.3 (2.9–42.8)	13.9 (3.0–42.8)	10.2 (2.9–33.8)	0.56
Lymphocytes (10^3^/μL)	1.2 (0.3–5.6)	1.1 (0.3–5.6)	1.4 (0.3–4.6)	0.59
N/L	8.6 (2.6–52.3)	9.4 (2.6–52.2)	8.2 (2.9–39.4)	0.22
RDW (%)	13.1 (10.9–20.8)	14.2 (11.9–20.8)	12.5 (10.9–19.8)	**0.01**
RDW/Ca	1.3 (1.1–2.4)	1.4 (1.1–2.4)	1.3 (1.1–2.2)	**0.03**
Platelets (10^3^/μL)	310 (34–777)	414 (34–742)	266 (106–777)	0.96
MPV (fL)	11.7 (8.5–25.9)	11.8 (8.9–24.4)	11.7 (8.5–25.9)	0.29
P/L	270 (13–1227)	325 (13–833)	234 (38.7–1227)	0.53

Significant values (*p* < 0.05) in bold characters. F, female; M, male; *n*, number of dogs; CRP, C-reactive protein; BUN, blood urea nitrogen; ALT, alanine aminotransferase; SAP, serum alkaline phosphatase; RBC, red blood cells; MCV, mean corpuscular volume; WBC, white blood cells; N/L, neutrophils-to-lymphocytes ratio; RDW, red cell distribution width; RDW/Ca, RDW-to-calcium ratio; P/L, platelets-to-lymphocytes ratio; MPV, mean platelet volume.

**Table 2:** Spearman correlation coefficients between red cell distribution width (RDW), RDW-to-calcium ratio (RDW/Ca), neutrophils-to-lymphocytes ratio (N/L) and platelets-to lymphocytes ratio (P/L), and laboratory variables.

Variable	RDW	*p* Value	RDW/Ca	*p* Value	N/L	*p* Value	P/L	*p* Value
CRP (mg/dL)	−0.202	0.10	−0.122	0.33	0.102	0.41	−0.079	0.52
BUN (mg/dL)	0.172	0.16	0.003	0.98	0.024	0.84	0.110	0.37
Creatinine (mg/dL)	0.156	0.20	−0.132	0.28	0.018	0.89	0.044	0.72
Protein (g/L)	0.176	0.14	−0.124	0.31	0.158	0.19	**0.330**	**0.01**
Albumin (g/L)	−0.028	0.82	−0.207	0.09	0.050	0.68	**0.304**	**0.01**
Bilirubin (mg/dL)	0.038	0.75	−0.006	0.96	0.074	0.54	0.085	0.48
Cholesterol (mg/dL)	0.010	0.93	−0.121	0.33	**0.245**	**0.04**	**0.346**	**<0.01**
Triglycerides (mg/dL)	−0.083	0.51	−0.147	0.25	**0.261**	**0.04**	0.064	0.61
Calcium (mg/dL)	0.061	0.62	**−0.658**	**<0.01**	−0.021	0.87	0.015	0.90
Phosphate (mg/dL)	**0.305**	**0.01**	0.054	0.66	0.229	0.06	0.117	0.34
Glucose (mg/dL)	**−0.254**	**0.04**	−0.098	0.43	0.028	0.82	**0.288**	**0.02**
ALT (U/L)	0.085	0.48	−0.056	0.65	0.211	0.08	0.047	0.70
SAP (U/L)	**0.264**	**0.03**	0.160	0.19	**0.347**	**<0.01**	**0.268**	**0.03**
RBC (10^6^/μL)	−0.225	0.05	−0.158	0.20	0.055	0.65	0.008	0.95
Hemoglobin (g/dL)	−0.217	0.07	−0.211	0.09	0.064	0.60	0.054	0.66
Hematocrit (%)	**−0.256**	**0.03**	−0.178	0.15	0.075	0.54	0.056	0.65
MCV (fL)	−0.027	0.82	−0.019	0.89	0.008	0.95	0.195	0.11
WBC (10^3^/μL)	**0.358**	**<0.01**	**0.268**	**0.03**	**0.471**	**<0.01**	−0.023	0.85
Neutrophils (10^3^/μL)	**0.352**	**<0.01**	**0.258**	**0.03**	**0.558**	**<0.01**	0.016	0.89
Lymphocytes (10^3^/μL)	0.021	0.86	0.022	0.86	**−0.516**	**<0.01**	**−0.542**	**<0.01**
Platelet (10^3^/μL)	0.085	0.49	0.080	0.52	0.173	0.15	**0.775**	**<0.01**
MPV (fL)	**0.255**	**0.03**	**0.284**	**0.02**	−0.109	0.35	**−0.405**	**<0.01**
RDW (%)	NA	NA	**0.650**	**<0.01**	**0.256**	**0.03**	0.035	0.77
RDW/Ca	**0.650**	**<0.01**	NA	NA	0.171	0.16	0.049	0.69
N/L	**0.256**	**0.03**	0.171	0.16	NA	NA	**0.467**	**<0.01**
P/L	0.035	0.77	0.049	0.69	**0.467**	**<0.01**	NA	NA

Significant correlation (*p* < 0.05) in bold characters. CRP, C-reactive protein; BUN, blood urea nitrogen; ALT, alanine aminotransferase; SAP, serum alkaline phosphatase; RBC, red blood cells; MCV, mean corpuscular volume; WBC, white blood cells; MPV, mean platelet volume; N/L, neutrophils-to-lymphocytes ratio; RDW, red cell distribution width; RDW/Ca, RDW-to-calcium ratio; P/L, platelets-to-lymphocytes ratio; NA, not applicable.

**Table 3:** Diagnostic accuracy of 4 laboratory variables to predict dead within 14 days in 70 dogs with acute pancreatitis.

Variable	AUC (95% CI)	*p* Value	Cut-Off	Sensitivity (95% CI)	Specificity (95% CI)
RDW (%)	0.74 (0.63–0.84)	<0.01	>12.7	89 (67–99)	53 (39–67)
RDW/Ca	0.67 (0.54–0.78)	0.03	>1.4	68 (43–87)	69 (55–82)
BUN (mg/dL)	0.70 (0.58–0.81)	<0.01	>42	79 (54–94)	62 (47–75)
RBC (10^6^/μL)	0.68 (0.55–0.78)	0.03	≤6.3	79 (54–94)	59 (44–72)

RDW, red cell distribution width; RDW/Ca, red cell distribution width-to-calcium ratio; BUN, blood urea nitrogen; RBC, red blood cell count; AUC, area under the curve; CI, confidence interval.

**Table 4:** Cox proportional univariate analysis for dead within 14 days in 70 dogs with acute pancreatitis.

Variable	Hazard Ratio	95% CI	Chi-Square	*p* Value	Q Value
BUN (mg/dL)	1.00	1.00–1.01	10.57	<0.01	**0.01**
Creatinine (mg/dL)	1.15	1.03–1.28	6.54	0.01	0.053
Bilirubin (mg/dL)	2.70	1.56–4.69	12.45	<0.01	**<0.01**
Phosphate (mg/dL)	1.10	1.03–1.17	7.76	<0.01	**0.03**
SAP (U/L)	1.00	1.00–1.00	4.66	0.03	0.078
RBC (10^6^/μL)	0.68	0.50–0.97	4.62	0.03	0.078
RDW (%)	1.22	1.04–1.43	6.09	0.01	0.054
RDW/Ca	4.49	1.27–15.89	5.43	0.02	0.065
RDW > 12.7%	7.14	1.65–30.93	6.90	<0.01	**0.04**
RDW/Ca > 1.4	3.75	1.42–9.90	7.14	<0.01	**0.04**

Significant correlations (Q < 0.05) for final multivariable model in bold characters. BUN, blood urea nitrogen; SAP, serum alkaline phosphatase; RBC, red blood cells; RDW, red cell distribution width; RDW/Ca, RDW-to-calcium ratio; CI, confidence interval.

**Table 5:** Results of the final multivariable model for dead within 14 days in 70 dogs with acute pancreatitis.

Predictors	Hazard Ratio	95% CI	Chi-Square	*p* Value
Bilirubin (mg/dL)	2.46	1.38–4.39	9.31	<0.01
BUN (mg/dL)	1.00	1.00–1.01	9.48	<0.01
RDW > 12.7%	5.08	1.14–22.67	4.52	0.03

BUN, blood urea nitrogen; RDW, red cell distribution width; CI: confidence interval.

## Data Availability

All data included in this study are available upon request by contacting the corresponding author.
